# Forward Bending in Supine Test: Diagnostic Accuracy for Acute Vertebral Fragility Fracture

**DOI:** 10.3390/healthcare10071215

**Published:** 2022-06-29

**Authors:** Chan-Woo Jung, Jeongik Lee, Dae-Woong Ham, Hyun Kang, Dong-Gune Chang, Youngbae B. Kim, Young-Joon Ahn, Joo Hyun Shim, Kwang-Sup Song

**Affiliations:** 1 Department of Orthopedic Surgery, Chung-Ang University Hospital, College of Medicine, Chung-Ang University, Seoul 06973, Korea; chanwoojungw@gmail.com (C.-W.J.); teddy9060@caumc.or.kr (J.L.); hamdgogo1@caumc.or.kr (D.-W.H.); 2 Department of Anesthesiology and Pain Medicine, Chung-Ang University Hospital, College of Medicine, Chung-Ang University, Seoul 06973, Korea; roman00@cau.ac.kr; 3 Department of Orthopedic Surgery, Sanggye Paik Hospital, College of Medicine, Inje University, Seoul 01757, Korea; dgchangmd@gmail.com; 4 Department of Orthopedic Surgery, VHS Medical Center, Seoul 05368, Korea; benspine@gmail.com; 5 Spine Center, Sesonneun Orthopedic Hospital, Seoul 04109, Korea; osahnyj@nate.com; 6 Department of Dermatology, National Police Hospital, Seoul 05715, Korea; drdr98@naver.com

**Keywords:** vertebral fracture, fragility fracture, physical examination, osteoporosis, diagnostic accuracy

## Abstract

Despite its high incidence rate, vertebral fragility fracture (VFF) is frequently underdiagnosed due to the absence of marked symptoms. This study evaluated the diagnostic accuracy of our suggested physical examinations and compared them with that of plain radiographs. Patients over 65 years of age with sudden back pain within the preceding 3 weeks were enrolled. Physical examinations in three different positions and a closed-fist percussion test were performed, and the presence of VFF was evaluated through confirmatory radiographic tools. We assessed the diagnostic accuracy of each physical examination and compared them with the interpretation of plain radiographs and examined the patient-reported pain locations based on the VFF level. A total of 179 patients were enrolled. The forward bending in supine (FB-SU) test demonstrated superior diagnostic values (sensitivity: 90.6%, specificity: 71.2%), which outperformed those of plain radiographs (sensitivity: 68.9%, specificity: 71.9%). The location of patient-reported pain was generally close to or lower than the index fracture level. FB-SU showed the highest diagnostic accuracy and was more valuable than plain radiographs in diagnosing acute VFF. FB-SU is a simple and affordable screening test. If positive, physicians should highly suspect VFF even when based on vague evidence of acute fracture provided by plain radiographs.

## 1. Introduction

Vertebral fragility fracture (VFF) is the most common type of osteoporotic fracture; its prevalence increases with age and can greatly vary worldwide [1]. A recent study reported that the prevalence of morphometric VFF can be as high as 26% to 34% in women ≥50 years old, according to race [2]. Despite this high prevalence, VFF has been underdiagnosed because it can present with rather mild or ambiguous initial symptoms and frequently occurs without a trauma history. Moreover, up to two-thirds of VFFs are not clinically diagnosed at the time of trauma [3]. As such, the suspicion of VFF can act as the first step toward preventing a delayed diagnosis and ensuring the appropriate time for osteoporosis treatment [4]. A neglected vertebral fracture might result in kyphotic deformity and nonunion, and these sequelae can be severely disabling to the affected individuals, leading to social and economic burdens on the community [5].

Plain radiographs have been in widespread use as a first-line tool to detect vertebral fracture; however, despite high specificity, they are known to have poor sensitivity [6]. The high variability between interpreters is another problem, with a false-negative rate of 34% being reported [7]. Furthermore, the correlation between symptoms and imaging findings is low, especially in patients with osteoporosis [8]. Computerized tomography (CT) and magnetic resonance imaging (MRI) can be confirmatory tools [9], but these are not always available, and there are cost and time constraints with conducting them for all patients visiting primary clinics.

With the increased incidence of VFF in an aging society, the suspicion of VFF based on patient history and focused physical examinations have been emphasized [10]. However, clinical suspicion itself is still not easy for physicians, especially for those who are not familiar with spine care, and there is a lack of data to identify reliable physical examination methods to discriminate the presence or absence of VFF [11].

This study aimed to investigate the diagnostic value of suggested physical examinations in three different positions, supine, sitting, and standing, for acute VFF, and to compare their accuracy to that of the plain radiograph, which is the most-used first diagnostic exam.

## 2. Materials and Methods

### 2.1. Study Participants

**Figure 1:**
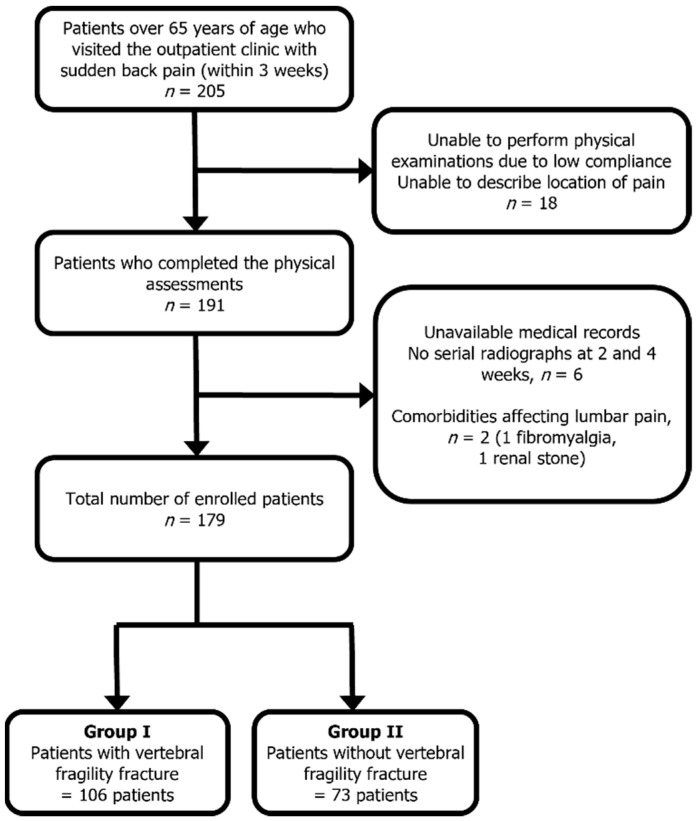
Flowchart of patient selection with inclusion and exclusion criteria.

This study was approved by the institutional review board and registered with the Clinical Research Information Service (KCT0002631). The population was prospectively enrolled by four independent institutions between November 2017 and November 2019. All participants were screened and evaluated for eligibility by individual authors before inclusion. The participants were persons over 65 years of age who complained of sudden-onset (within the past 3 weeks) back pain with or without minor trauma, corresponding to the definition of fragility fracture (which would not normally result in a fracture, such as a fall from a standing height or less [12]) in the outpatient clinic of each hospital. Patients who were associated with a traffic accident, high-energy trauma, back pain treated with medication in the last 3 months, and other comorbidities affecting back pain were excluded (Figure 1). Demographic data including age, body mass index (BMI), and bone mineral density (BMD) were investigated [13].

### 2.2. Physical Examinations

**Figure 2:**
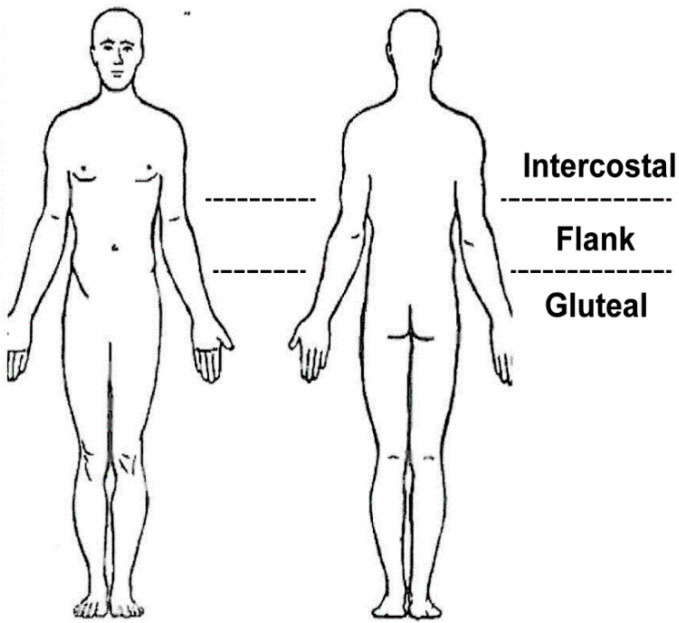
Illustration of patient-drawn pain locations on the human figure, divided into intercostal, flank (12th rib to iliac bone), and gluteal areas.

All physical examinations were performed at the first clinical visit. The location of the pain and tenderness area were documented. Patient-drawn pain locations on the human figure diagram (Figure 2) were divided into intercostal, flank (12th rib to iliac bone), and gluteal areas. Tenderness was examined with a closed-fist percussion sign, which required the examiner to stand behind the seated patient and evaluate the entire length of the spine using firm, closed-fist percussions [14].

**Figure 3:**
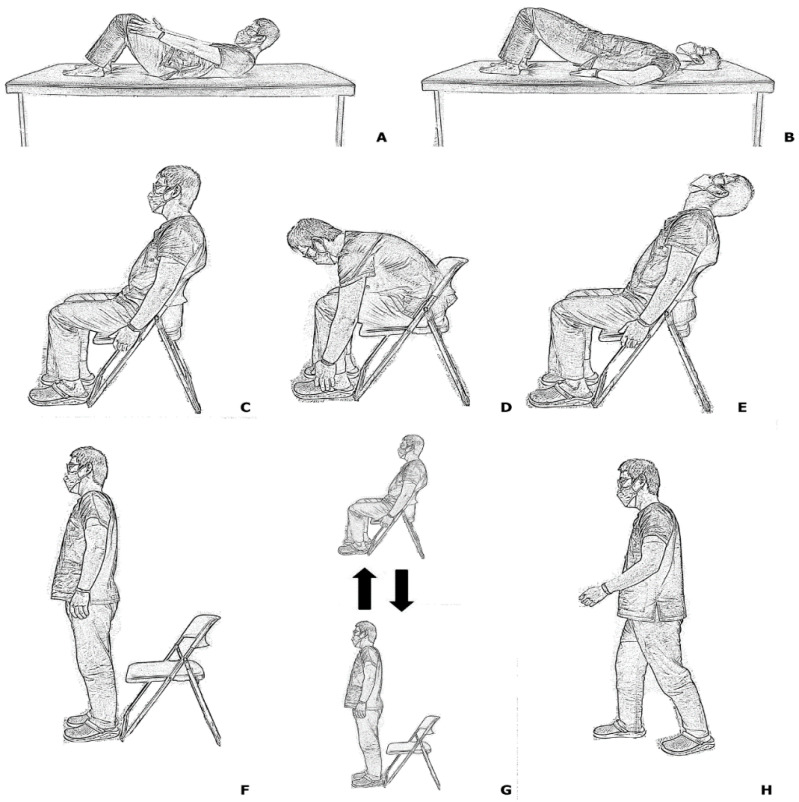
Illustration of proposed physical examinations: (**A**) forward bending in supine (FB-SU), and (**B**) backward bending in supine (BB-SU); (**C**) sitting for 5 s (SIT), (**D**) forward bending in sitting (FB-SI), and (**E**) backward bending in sitting (BB-SI); (**F**) standing for 5 s (STAND), (**G**) sitting to standing, standing to sitting (SIT-STAND), and (**H**) walking for 10 s (WALK).

Then, eight index physical examinations were serially performed in three different positions. The supine position, forward bending in supine (FB-SU), and backward bending in supine (BB-SU) tests were performed. While performing the FB-SU test, the patient was instructed to slightly bend their back forward and attempt to touch areas around their knee with both hands. In the BB-SU test, the patient was instructed to lift their buttock slightly, just enough to slide the examiner’s hands in the area. Then, the patient was positioned in a sitting position; and sitting for 5 s (SIT), forward bending in sitting position (FB-SI), and backward bending in sitting position (BB-SI) tests were performed. The FB-SI test required the patient to bend forward and touch the dorsum of their feet, while the BB-SI test required the patient to bend the back backward and gaze at the ceiling. Finally, the patient was instructed to stand up and perform standing for 5 s (STAND), sitting to standing, standing to sitting (SIT-STAND), and walking 10 steps (WALK) (Figure 3). If the patients were able to perform each index physical examination, the result was deemed negative; however, if the patients could not perform due to pain or discomfort, the result was deemed positive.

The presence of acute VFF was evaluated through confirmatory radiographic tools (serial radiographs, CT, and MRI). All patients were examined with plain radiographs. If the patient underwent CT or MRI, acute VFF was immediately diagnosed if CT or MRI examinations were unavailable, the patients were subjected to a serial plain radiograph at 2 and 4 weeks of follow-up. If further morphometric collapse or sclerotic changes on suspected vertebra were noted, the diagnosis for acute VFF was confirmed in each institution.

### 2.3. Evaluation of Plain Radiograph

Initial radiographs of all patients were provided to two independent evaluators (rater 1: orthopedic spine surgeon with 5 years of experience; rater 2: 3rd-year orthopedic resident). A patient-drawing diagram regarding pain location was provided with the radiograph, and the interpretation of the presence and location of acute VFF was recorded. This interpretation process was repeated twice at two-week intervals for both evaluators. The acute fracture levels were divided into 3 groups: thoracic, thoracolumbar (T11 to L1), and lumbar spines.

### 2.4. Statistical Analysis

The sample size was based on the result of a previous study that reported a fracture prevalence of 55.8% (67 fracture cases out of 120 patients), a sensitivity of 55.5%, and a discordance rate between MRI and plain radiograph of 48.5% [15]. Under the assumption that physical examination results improved sensitivity to 81.5%, with the fracture proportion in the study group being 60%, the necessary sample size was 174 cases with α = 0.05 and a power of 80%. Presuming a dropout rate of 15%, we decided to enroll a total of 205 patients. As a diagnostic accuracy study, we followed Standards for Reporting Diagnostic Accuracy Studies 2015 guidelines [16]. We calculated each physical examination’s sensitivity, specificity, and accuracy using a 2 × 2 contingency table for diagnosing fragility fracture. The McNemar test was conducted to evaluate whether the difference between diagnostic examinations was statistically significant [17]. Furthermore, the McNemar test was used to evaluate the difference between confirmatory diagnosis and each diagnostic examination, and that between the plain radiograph and each diagnostic examination.

Descriptive statistics were used for group comparisons. The demographic data and their statistical significance were compared between fracture and non-fracture groups and were analyzed with a two-sample t-test using SPSS ver. 19.0 software (IBM/SPSS; Armonk, New York, NY, USA). A *p*-value < 0.05 was considered statistically significant. The reliability of radiographic interpretation for the presence of acute VFF was evaluated with kappa values, and the strength of agreement was assessed with the kappa value agreement range as defined by Cohen [18].

## 3. Results

### 3.1. Demographics

A total of 205 patients were first enrolled. Of these, 18 patients who were unable to perform all the physical examinations, 2 patients with a previous diagnosis of fibromyalgia and renal stone, and 6 patients without confirmatory radiographic results were excluded. Finally, 179 patients were enrolled in our study and divided into the fracture group (*n* = 106) and the non-fracture group (*n* = 73) (Figure 1). The mean age of the patients was 73.96 years (95% CI: 72.84–75.08), of which 35 were men and 144 were women. Demographic data including age, height, weight, BMI, and BMD (lumbar, femur neck, and hip total) demonstrated no significant differences between the fracture and non-fracture groups. We evaluated 74 patients for the presence of acute VFF with serial radiographs, 23 patients with CT, and 82 patients with MRI (Table 1).

### 3.2. Diagnostic Values of Physical Examinations

**Figure 4:**
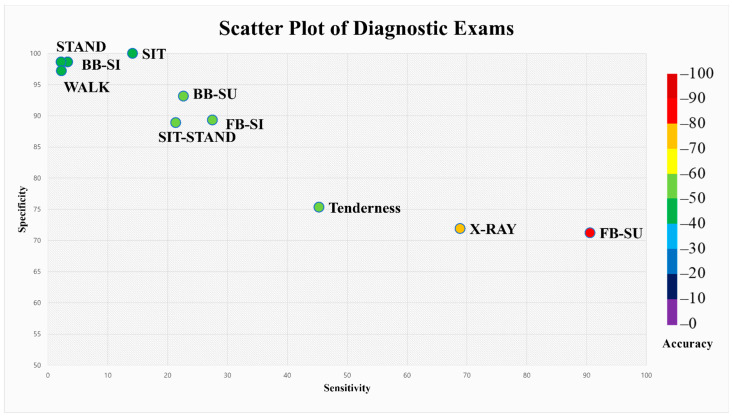
Scatter plot diagram of physical examinations and radiographic interpretations illustrating sensitivity, specificity, and accuracy.

The sensitivity, specificity, and accuracy were evaluated for the eight index physical examinations, as shown in Table 2, and their comparative values are demonstrated in scatter plot diagrams (Figure 4). Cross-tabulation diagrams of the physical examinations are provided in Table 3.

The FB-SU test demonstrated superior diagnostic value compared with the other physical examinations, with a sensitivity of 90.6%, specificity of 71.2%, and accuracy of 82.7%. Plain radiographic interpretation of acute VFF with clinical symptoms had diagnostic value with a sensitivity of 68.9%, specificity of 71.9%, and accuracy of 70.12%, thereby underperforming against the accuracy of FB-SU by more than 10%. Other physical examinations, including BB-SU, SIT, FB-SI, BB-SI, STAND, SIT-STAND, and WALK, demonstrated extremely low sensitivity values ranging from 14.2% to 37.7% and, in contrast, showed higher specificity values ranging from 87.7% to 100%. In the case of tenderness, which is the physical examination most frequently performed in clinics, the sensitivity, specificity, and accuracy were 45.3%, 75.3%, and 57.5%, respectively.

The McNemar test results revealed that only FB-SU and plain radiographic interpretations by rater 2 demonstrated significant diagnostic values (*p* > 0.025) compared with the confirmatory results. Additionally, the McNemar test results between physical examinations and plain radiographic interpretations by rater 2 showed that FB-SU was a more valuable diagnostic tool, having a higher sensitivity value of 90.6% compared with that of rater 1 at 69.3% (Table 2). No significant or minor adverse events occurred as a result of the suggested physical examinations.

### 3.3. Reliability of Plain Radiograph

The intra-rater reliability of rater 1 showed a kappa value of 0.777 (substantial agreement), while that of rater 2 showed 0.552 (moderate agreement). The inter-rater reliability between the two raters demonstrated a kappa value of 0.474, which indicated moderate strength of agreement.

### 3.4. Location of Patient-Drawing Pain Based on Fracture Level

**Figure 5:**
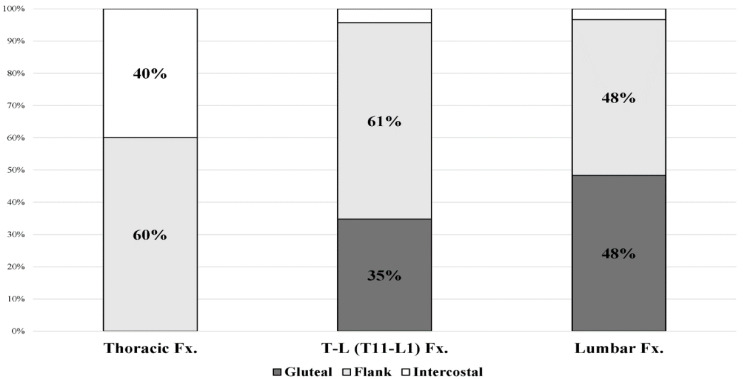
Paraspinal pain distribution according to vertebral fracture level. T-L: thoraco-lumbar.

In the case of thoracic fracture, the most frequent location of pain was the flank area, and thoracolumbar and lumbar fractures showed that the location of pain was mostly in the flank and gluteal areas. Upon analysis of the location of paraspinal pain correlated with fracture levels, the pain was largely located distal to the fracture level (flank area in 60% of thoracic fractures, the gluteal area in 35% of thoracolumbar fractures, and 48% of lumbar fractures (Figure 5)).

## 4. Discussion

This multicenter study aimed to evaluate the diagnostic accuracy of suggested physical examinations for acute VFF. FB-SU had the most reliable diagnostic accuracy for acute VFF among the suggested physical examinations, which was higher than those of plain radiographs, which are thought to be an appropriate first-line screening test in suspecting vertebral fracture by most physicians.

The ideal screening test should be reproducible, cost-effective, non-invasive, and have high sensitivity and specificity. A test with both sensitivity and specificity close to 90% is generally regarded as having acceptable diagnostic performance [19], but high sensitivity may be desired over specificity, especially when the disease is severe and intervention at an early clinical stage is beneficial [20]. Considering the requirements as a diagnostic test, FB-SU can be sufficiently applied as a preliminary diagnostic test for acute VFF.

The other seven suggested physical examinations, compared with FB-SU, showed higher specificity but much lower sensitivity, which means that the majority of patients with VFFs can perform these examinations without severe pain interrupting the examinations. Although various factors can be responsible, the most probable factor might be the stability around the fractured vertebra by guarding paravertebral musculatures, these muscle contractions significantly decreasing the motion around the fracture site [21]. The forward-bending postures in our study required greater change in compressive motion than backward-bending postures, and appeared to be less guarded by the surrounding musculatures. Therefore, backward-bending postures (BB-SU and BB-SI), whether in the supine or sitting position, can mask the pain due to the abovementioned guarding of muscles. Static spine postures, such as SIT, STAND, and WALK, can be similarly explained and does not provide motion stress at fractured vertebra from a change in position. The FB-SI and SIT-STAND tests showed relatively higher sensitivity compared with other examinations except for FB-SU. These tests may be prone to greater change in position, especially in flexion posture. However, compared with the FB-SU test, they can be performed in conditions of relative back extension using a support, such as a chair or bed, with both hands. In contrast, the FB-SU test induces flexion stress in the supine position by the requirement of touching both hands to the area around the knee, and it is considered to increase the sensitivity to pain. During the patient interview, most of the patients with VFF could not immediately rise in the supine position and had to rise following turning to the side due to pain, which is another illustration of a situation in which pain is most likely to occur. Postacchini et al. [22] suggested that certain physical examinations such as sitting, lying supine, and turning around on the back elicit pain-related behavior, which are evident enough to suspect, or even diagnose, the presence of a vertebral fracture with an accuracy of 80–87%, which is similar to that of FB-SU (82.7%). However, it is difficult to identify the behavior responsible for pain because authors have comprehensively judged pain using only continuous videotaped motion, and a small sample size with different times of injury might be a confounding factor. Thus, the rationale for our physical examinations was to divide these continuous pain-provoking motions into specific positions and movements, such as FB-SU, and to identify their independent effectiveness in detecting and diagnosing vertebral fragility fracture. Although the pain-provocation mechanism may be multifactorial, VFF not only damages the vertebral body but also subsequently affects the surrounding structures associated with pain generation [23]. Changes in pressure on disrupted endplates during flexion movement, causing irritation on structures such as the mechanosensitive basivertebral nerve, can be considered the main cause, which is consistent with our results that FB-SU has the highest diagnostic values [24].

One of the widely used physical examinations for vertebral fracture is the percussion test. However, it showed a relatively low diagnostic accuracy that was similar to that reported in previous studies, suggesting that a lack of focal midline tenderness does not exclude vertebral fracture [25]. Furthermore, the quality of the focal tenderness exam substantially varied depending on the examiner, because the percussion or compression strength is difficult to standardize [26].

As for the diagnostic value of plain radiographs, unsatisfactory and varying diagnostic values for vertebral fracture (sensitivity: 7.7 to 86%, specificity: 95 to 100%) were previously demonstrated in the thoracic and lumbar spine [6]. Our results demonstrated that the sensitivity and specificity values were 68–69% and 72%, respectively. The diagnostic value, as recorded by rater 2, was comparable to that of confirmatory diagnosis (*p* = 0.026), but FB-SU revealed a higher diagnostic value and higher sensitivity according to the results of the McNemar test (Table 2). In addition, inter-rater reliability analysis revealed moderate agreement, suggesting that plain radiograph interpretation may be inconsistent between physicians of varying clinical experience.

Patient-drawing pain locations demonstrated that paraspinal pain generally occurred close to or lower than the index fracture site, suggesting that sudden sharp motion pain, especially in flexion of the spine, may be caused by both direct distortions of bone innervating the mechanosensitive nerve fibers and irritation of the sinuvertebral nerve [27]. In contrast, diffuse paravertebral pain below or adjacent to the fracture site may be caused by mechanical stress on the facet joint or strain of the paraspinal muscles, which are innervated by posterior branches of the spinal nerves [28].

Our study had some limitations. First, our data included only patients who met the criteria for the injury mechanism of fragility fracture, and who walked into the outpatient clinics on their own, while excluding patients with acute vertebral fracture who visited the emergency department with severe pain. This makes our results inapplicable to all vertebral fractures. Second, there was a possibility that minor fracture cases that do not cause substantial vertebral body collapse could have been miscategorized as the non-fracture group. However, in most cases of VFF, even linear fractures, morphometric changes in the vertebral body appear within a few days to weeks [29]. As such, incorrect categorization was considered unlikely. Third, the pain scale was not analyzed in the present study, and patients who were able to perform the examination despite severe pain could not be separately classified. Lastly, patients may have had a different degree of tolerance to pain, which might have resulted in false-positive or false-negative results in a few cases. We think that numerical pain scales should be included in future studies for further classification and evaluation.

## 5. Conclusions

In conclusion, the FB-SU test had the most reliable diagnostic accuracy among other physical findings, and the values were higher than those of plain radiographs in diagnosing acute VFF. FB-SU is affordable and simple, even if physicians are not particularly familiar with spine care. We recommend the test for patients over 65 years old complaining of acute back pain within 3 weeks after symptom onset. If positive, the physician should highly suspect the possibility of VFF even when lacking evidence of acute fracture provided by plain radiographs.

## Figures and Tables

**Table 1:** Demographic data of fracture and non-fracture groups.

Parameter	Fracture (*n* = 106)	Non-Fracture (*n* = 73)	*p*-Value
Age	74.84 (±7.45)	72.68 (±7.44)	0.065
Height (cm)	156.38 (±9.09)	155.83 (±8.63)	0.683
Weight (kg)	57.34 (±10.25)	58.54 (±10.92)	0.461
BMI	23.68 (±5.16)	23.70 (±5.18)	0.548
Lumbar BMD (g/cm^2^)	0.90 (±0.18)	0.91 (±0.17)	0.784
Femur neck BMD (g/cm^2^)	0.67 (±0.13)	0.67 (±0.12)	0.792
Hip total BMD (g/cm^2^)	0.71 (±0.14)	0.72 (±0.72)	0.591
Diagnostic modality			
Serial radiograph/CT/MRI	23/14/69	51/9/13	
Fracture location			
Thoracic/Thoracolumbar/Lumbar	10/33/63		

BMI: body mass index, BMD: bone mineral density, CT: computerized tomography, and MRI: magnetic resonance imaging.

**Table 2:** Diagnostic values of physical examinations and radiograph interpretations.

Diagnostic Test	Sensitivity (%)	Specificity (%)	Accuracy (%)	McNemar Test *p*-Value *	
				Compared with Confirmatory Results	Compared with Radiographic Results of Rater 2
FB-SU	90.6	71.2	82.7	0.072	<0.001
BB-SU	22.6	93.2	51.4	<0.001	<0.001
SIT	14.2	100.0	49.2	<0.001	<0.001
FB-SI	37.7	91.8	59.7	<0.001	<0.001
BB-SI	17.0	98.6	50.3	<0.001	<0.001
STAND	16.0	98.6	49.7	<0.001	<0.001
SIT-STAND	34.0	87.7	55.9	<0.001	<0.001
WALK	17.9	95.9	49.7	<0.001	<0.001
Tenderness	45.3	75.3	57.5	<0.001	<0.001
Radiographic interpretation (rater 1)	68.4	71.9	69.8	0.016	-
Radiographic interpretation (rater 2)	69.3	71.9	70.4	0.026	-

FB-SU: forward bending in supine, BB-SU: backward bending in supine, SIT: sitting for 5 s, FB-SI: forward bending in sitting, BB-SI: backward bending in sitting, STAND: standing for 5 s, SIT-STAND: sitting to standing, standing to sitting, and WALK: walking for 10 s; * McNemar *p*-value greater than 0.05 indicates that the examination had diagnostic power statistically equivalent to that of the confirmatory test. For each rater, radiographic interpretation was performed twice, so *p*-value greater than 0.025 was statistically equivalent.

**Table 3:** Cross-tabulation (2 × 2) diagram of index physical examinations.

**FB-SU**	Fracture (+)	Fracture (−)	Total	**BB-SU**	Fracture (+)	Fracture (−)	Total
Finding (+)	96	21	117	Finding (+)	24	5	29
Finding (−)	10	52	62	Finding (−)	82	68	150
Total	106	73	179	Total	106	73	179
**SIT**				**FB-SI**			
Finding (+)	15	0	15	Finding (+)	40	6	46
Finding (−)	91	73	164	Finding (−)	66	67	133
Total	106	73	179	Total	106	73	179
**BB-SI**				**STAND**			
Finding (+)	18	1	19	Finding (+)	17	1	18
Finding (−)	88	72	160	Finding (−)	89	72	161
Total	106	73	179	Total	106	73	179
**SIT-STAND**				**WALK**			
Finding (+)	36	9	45	Finding (+)	19	3	22
Finding (−)	70	64	134	Finding (−)	**87**	70	157
Total	106	73	179	Total	106	73	179
**Tenderness**							
Finding (+)	48	18	66				
Finding (−)	58	55	113				
Total	106	73	179				

FB-SU: forward bending in supine, BB-SU: backward bending in supine, SIT: sitting for 5 s, FB-SI: forward bending in sitting, BB-SI: backward bending in sitting, STAND: standing for 5 s, SIT-STAND: sitting to standing, standing to sitting, and WALK: walking for 10 s.

## Data Availability

The data presented in this study are available on request from the corresponding author. The data are not publicly available due to ethical and privacy reasons.

## References

[1] Curtis E.M., Moon R.J., Harvey N.C., Cooper C. (2017). Reprint of: The impact of fragility fracture and approaches to osteoporosis risk assessment worldwide. Int. J. Orthop. Trauma Nurs..

[2] Ballane G., Cauley J.A., Luckey M.M., El-Hajj Fuleihan G. (2017). Worldwide prevalence and incidence of osteoporotic vertebral fractures. Osteoporos. Int..

[3] Kendler D.L., Bauer D.C., Davison K.S., Dian L., Hanley D.A., Harris S.T., McClung M.R., Miller P.D., Schousboe J.T., Yuen C.K. (2016). Vertebral Fractures: Clinical Importance and Management. Am. J. Med..

[4] El Maghraoui A., Rezqi A., Mounach A., Achemlal L., Bezza A., Ghozlani I. (2013). Systematic vertebral fracture assessment in asymptomatic postmenopausal women. Bone.

[5] Suzuki N., Ogikubo O., Hansson T. (2008). The course of the acute vertebral body fragility fracture: Its effect on pain, disability and quality of life during 12 months. Eur. Spine J..

[6] Gehlbach S.H., Bigelow C., Heimisdottir M., May S., Walker M., Kirkwood J.R. (2000). Recognition of vertebral fracture in a clinical setting. Osteoporos. Int..

[7] Lee Y.W., Jang J.H., Kim J.J., Lim Y.S., Hyun S.Y., Yang H.J. (2017). The Value of X-ray Compared with Magnetic Resonance Imaging in the Diagnosis of Traumatic Vertebral Fractures. J. Trauma Inj..

[8] Delmas P.D., van de Langerijt L., Watts N.B., Eastell R., Genant H., Grauer A., Cahall D.L. (2005). Underdiagnosis of Vertebral Fractures Is a Worldwide Problem: The IMPACT Study. J. Bone Miner. Res..

[9] Hirsch J.A., Beall D.P., Chambers M.R., Andreshak T.G., Brook A.L., Bruel B.M., Deen H.G., Gerszten P.C., Kreiner D.S., Sansur C.A. (2018). Management of vertebral fragility fractures: A clinical care pathway developed by a multispecialty panel using the RAND/UCLA Appropriateness Method. Spine J..

[10] Clark E.M., Cummings S.R., Schousboe J.T. (2017). Spinal radiographs in those with back pain-when are they appropriate to diagnose vertebral fractures?. Osteoporos. Int..

[11] Longo U.G., Loppini M., Denaro L., Maffulli N., Denaro V. (2011). Osteoporotic vertebral fractures: Current concepts of conservative care. Br. Med. Bull..

[12] Compston J., Cooper A., Cooper C., Gittoes N., Gregson C., Harvey N., Hope S., Kanis J.A., McCloskey E.V., Poole K.E.S. (2017). UK clinical guideline for the prevention and treatment of osteoporosis. Arch. Osteoporos..

[13] Lee J., Chang G., Kang H., Ham D.-W., Lee J.-S., Jung H.S., Song K.-S. (2020). Impact of Bone Mineral Density on the Incidence of Age-Related Vertebral Fragility Fracture. J. Korean Med. Sci..

[14] Langdon J., Way A., Heaton S., Bernard J., Molloy S. (2010). Vertebral compression fractures—New clinical signs to aid diagnosis. Ann. R. Coll. Surg. Engl..

[15] Ito Z., Harada A., Matsui Y., Takemura M., Wakao N., Suzuki T., Nihashi T., Kawatsu S., Shimokata H., Ishiguro N. (2006). Can you diagnose for vertebral fracture correctly by plain X-ray?. Osteoporos. Int..

[16] Cohen J.F., Korevaar D.A., Altman D.G., Bruns D.E., Gatsonis C.A., Hooft L., Irwig L., Levine D., Reitsma J.B., de Vet H.C.W. (2016). STARD 2015 guidelines for reporting diagnostic accuracy studies: Explanation and elaboration. BMJ Open.

[17] Lachenbruch P.A., Balakrishnan N., Colton T., Everitt B., Piegorsch W., Ruggeri F., Teugels J.L. (2014). McNemar Test. Wiley StatsRef: Statistics Reference Online.

[18] Fleiss J.L., Cohen J. (1973). The Equivalence of Weighted Kappa and the Intraclass Correlation Coefficient as Measures of Reliability. Educ. Psychol. Meas..

[19] Šimundić A.-M. (2009). Measures of Diagnostic Accuracy: Basic Definitions. Electron. J. Int. Fed. Clin. Chem. Lab. Med..

[20] Herman C.R., Gill H.K., Eng J., Fajardo L.L. (2002). Screening for Preclinical Disease: Test and Disease Characteristics. Am. J. Roentgenol..

[21] Briggs A.M., Greig A.M., Bennell K.L., Hodges P.W. (2007). Paraspinal muscle control in people with osteoporotic vertebral fracture. Eur. Spine J..

[22] Postacchini R., Paolino M., Faraglia S., Cinotti G., Postacchini F. (2013). Assessment of patient’s pain-related behavior at physical examination may allow diagnosis of recent osteoporotic vertebral fracture. Spine J..

[23] Doo T.-H., Shin D., Kim H.-I., Shin D.-G., Kim H.-J., Chung J.-H., Lee J.-O. (2009). Clinical Relevance of Pain Patterns in Osteoporotic Vertebral Compression Fractures. J. Korean Med. Sci..

[24] Fischgrund J.S., Rhyne A., Macadaeg K., Moore G., Kamrava E., Yeung C., Truumees E., Schaufele M., Yuan P., De Palma M. (2020). Long-term outcomes following intraosseous basivertebral nerve ablation for the treatment of chronic low back pain: 5-year treatment arm results from a prospective randomized double-blind sham-controlled multi-center study. Eur. Spine J..

[25] D’Costa H., George G., Parry M., Pullinger R., Skinner D., Thomas S., Todd B., Wilson M. (2005). Pitfalls in the clinical diagnosis of vertebral fractures: A case series in which posterior midline tenderness was absent. Emerg. Med. J..

[26] Nolet P.S., Yu H., Côté P., Meyer A.-L., Kristman V.L., Sutton D., Murnaghan K., Lemeunier N. (2021). Reliability and validity of manual palpation for the assessment of patients with low back pain: A systematic and critical review. Chiropr. Man. Ther..

[27] Will J.S., Bury D.C., Miller J.A. (2018). Mechanical Low Back Pain. Am. Fam. Phys..

[28] Choi Y.-S. (2009). Pathophysiology of degenerative disc disease. Asian Spine J..

[29] Old J.L., Calvert M. (2004). Vertebral compression fractures in the elderly. Am. Fam. Phys..

